# Childbirth Experiences and Challenges for Women with Sensory Disabilities: A Systematic Review of Delivery Methods and Healthcare Barriers

**DOI:** 10.34763/jmotherandchild.20242801.d-24-00038

**Published:** 2025-02-11

**Authors:** Daniela Sula, Chrysoula Rozalia Athanasiadou, Dimitra Metallinou, Kleanthi Gourounti, Antigoni Sarantaki

**Affiliations:** Department of Midwifery, School of Health and Care Science, University of West Attica (UNIWA), Egaleo, Athens, Greece

**Keywords:** sensory issues, women with disability, modes of birth, caesarean section, midwifery, delivery

## Abstract

**Background:**

Women with sensory disabilities, including deafness and blindness, face significant barriers to equitable healthcare in pregnancy, childbirth, and postnatal care. Representing over 5% of the global population—a number expected to rise—these women often encounter discrimination, limited information access, and inadequate childbirth support, increasing pregnancy-related risks.

**Materials and methods:**

This systematic review examines childbirth methods for women with sensory disabilities and the healthcare barriers they face during prenatal, perinatal, and postnatal periods. Using the Preferred Reporting Items for Systematic Reviews and Meta-Analyses (PRISMA) 2020 methodology, searches were performed in PubMed, Scopus, BioMed Central, and Cochrane Library databases. From 270 relevant studies, 10 met the inclusion criteria, comprising 8 quantitative and 2 qualitative studies. All studies were critically appraised using the Caldwell framework.

**Results:**

The review identified that women with sensory disabilities, particularly those who are deaf or blind, experience higher rates of caesarean sections compared to women without disabilities. However, a significant proportion of women in this demographic group successfully deliver vaginally. The review also highlighted substantial healthcare barriers, including inadequate communication between patients and healthcare providers, limited information regarding childbirth options, and insufficient postnatal care. Discrimination and obstetric violence were reported in several studies, further exacerbating the healthcare experiences of these women.

**Conclusions:**

This study highlights the urgent need for healthcare systems to enhance communication, accessibility, and support for women with sensory disabilities. An equity and inclusion framework in maternal care should ensure that these women receive adequate and respectful healthcare. Addressing these gaps will improve outcomes for mothers and newborns and reduce discrimination and inequitable treatment.

## Introduction

Women with sensory disabilities, including hearing loss and visual impairments, represent a significant portion of the global population. According to estimates from the World Health Organization (WHO), this demographic comprises over 5% of the world’s population, equating to approximately 430 million individuals. This figure is projected to exceed 700 million by 2050, reflecting the growing importance of addressing the unique challenges faced by this group in various aspects of healthcare, including childbirth (1).

Despite advancements in healthcare, women with disabilities continue to face numerous medical and socio-economic challenges that impact their pregnancy and childbirth experiences. Medically, these challenges are often linked to the nature of their disabilities and associated physiological issues. Socio-economically, barriers include limited access to education, poverty, discrimination, and a lack of inclusion within healthcare systems, all of which contribute to higher risks during pregnancy, childbirth, and the postpartum period (2). To address these inequities, the WHO has called for the establishment of an inclusive healthcare framework that aims to ensure that women with disabilities receive equitable and adequate care, thereby safeguarding both the mothers and their newborns from adverse health outcomes and social discrimination (1, 2).

A notable concern in maternal care for women with sensory disabilities is the increased likelihood of caesarean section deliveries. Caesarean sections, while often medically necessary, are associated with longer recovery times and potential negative psychological effects for the mother (3,4). Despite these risks, the global rate of caesarean deliveries continues to rise, currently accounting for 21% of all births worldwide, with projections suggesting that this figure could approach 29% by 2030 (1). This trend raises critical questions about whether women with sensory disabilities are disproportionately subjected to interventional deliveries, such as caesarean sections, and whether they experience limitations in exercising autonomy over their childbirth choices (5, 6).

This article aims to systematically review the existing literature on childbirth experiences of women with sensory disabilities, specifically focussing on their rates of caesarean section and other interventional birth methods. Additionally, it seeks to identify the healthcare barriers these women face and offer recommendations to improve maternal care services. This review will contribute to ongoing discussions on reproductive rights, equality in healthcare, and the mental health impacts of childbirth on women with sensory disabilities.

## Materials and methods

### Search strategy

A comprehensive search strategy was employed to identify relevant literature for this systematic review, focussing on childbirth experiences and healthcare barriers for women with sensory disabilities. The search was conducted across several key scientific databases, including PubMed/Medline, Scopus, BioMed Central, and the Cochrane Library. These databases were selected for their extensive coverage of medical and healthcare research.

Initially, a broad search was performed using a wide array of relevant terms. This strategy was subsequently refined, narrowing the scope to two specific search algorithms. The terminology included keywords related to midwifery, childbirth, pregnancy, and women with sensory disabilities, such as deaf or blind women. The search adhered to the guidelines of the Preferred Reporting Items for Systematic Reviews and Meta-Analyses (PRISMA) statement, ensuring a transparent and systematic approach to the literature review and source citation (7).

The two search algorithms employed in this study are detailed in Table 1, which outlines the database, search terms, and the corresponding research questions addressed by each algorithm. This systematic review was registered with the International Prospective Register of Systematic Reviews (PROSPERO) under the registration number CRD42024593330.

**Table 1.** Research algorithms.

**Number of the Algorithm**	**Database**	**Search Algorithm**	**Research Question**
**1.**	PubMed / Medline και BioMed Central	(“**birth**” OR “**labor**” OR “**labour**” OR “**delivery**” OR “**vaginal-birth**” OR “**vaginal-birthing**” OR “**vaginal-birth-after-caesarean**” OR “**VBAC**” OR “**c-section**” OR “**home birth**” OR “**water birth**” OR “**waterbirthing**” OR “**homebirthing**” OR “**childbirth**” OR “**parturition**” OR “**giving-birth**” OR “**natural-delivery**” OR “**natural-birth**” OR “**accouchement**” OR “**natural-childbirth**” OR “**birthing**” OR “**Caesarean-section**” OR “**confinement”**) **AND** (“**deaf-mothers**” OR “**deaf-mother**” OR “**hearing-loss-mothers**” OR “**deafness-mothers**” OR “**hearing-loss-mother**” OR “**deafness-mother**” OR “**Deaf-women**” OR “**hearing-loss-women**” OR “**deafness-women**” OR “**Deaf-woman** “**hearing-loss-woman**” OR “**deafness-woman**” OR “**sensory-disabilities-mothers**” OR “**sensory-disabilities-mother**” OR “**blind-mothers**” OR “**blind-mother**” OR “**blind-women**” OR “**sensory-disabilities-women**”)	Type of childbirth for women with sensory disability (deaf or / and blind).
**2.**	Scopus and Cochrane Library	(“**birth**” OR “**labor**” OR “**delivery**” OR “**vaginal birth**” OR “**vaginal birthing**” OR “**vaginal birth after caesarean**” OR “**VBAC**” OR “**c section**” OR “**home birth**” OR “**water birth**” OR “**waterbirthing**” OR “**homebirthing**” OR “**childbirth**” OR “**parturition**” OR “**giving birth**” OR “**natural delivery**” OR “**natural birth**” OR “**accouchement**” OR “**natural childbirth**” OR “**birthing**” OR “**Caesarean section**” OR “**confinement**” OR “**csection”**) **AND** (“**deaf mothers**” OR “**deaf mother**” OR “**hearing loss mothers**” OR “**deafness mothers**” OR “**hearing loss mother**” OR “**deafness mother**” OR “**Deaf women**” OR “**hearing loss women**” OR “**deafness women**” OR “**Deaf woman**” OR “**hearing loss woman**” OR “**deafness woman**” OR “**sensory disabilities mothers**” OR “**sensory disabilities mother**” OR “**blind mothers**” OR “**blind mother**” OR “**blind women**” OR “**sensory disabilities women**”)	Type of childbirth for women with sensory disability (deaf or / and blind).

### Criteria for inclusion and exclusion

The inclusion and exclusion criteria for this review were guided by the PICOST framework (8), a tool that ensures a structured and transparent selection process.

The criteria were applied as follows: (a) Population: The studies included were focussed on women with sensory disabilities who were pregnant or had given birth; (b) Intervention: The focus was on the types of childbirth experienced by women with sensory disabilities; (c) Comparison: Comparative analysis was conducted on the types of childbirth for women with sensory disabilities versus those without such disabilities; (d) Outcome: The primary outcomes included the method of delivery and associated healthcare barriers; (e) Study: Only primary research studies (quantitative and qualitative) were included. Articles not available in full text or written in languages other than English were excluded; and (f) Timeliness: Studies published between January 1, 2010, and April 24, 2024, were considered.

### PRISMA process

The initial search across the databases identified a total of 270 entries. Following the removal of five duplicate entries, 265 unique records remained for further evaluation. Titles and abstracts were screened to exclude studies not directly related to the research objective, focussing specifically on childbirth experiences for women with sensory disabilities (deaf, blind, or otherwise).

After the screening process, 254 articles were excluded, leaving 11 articles for further review. Of these, three articles were inaccessible, and one was excluded for being in a language other than English. This resulted in a final sample of seven articles. Additionally, 3 relevant studies were added, bringing the total number of articles analysed to 10, comprising 8 quantitative studies and 2 qualitative studies.

**Figure 1.**
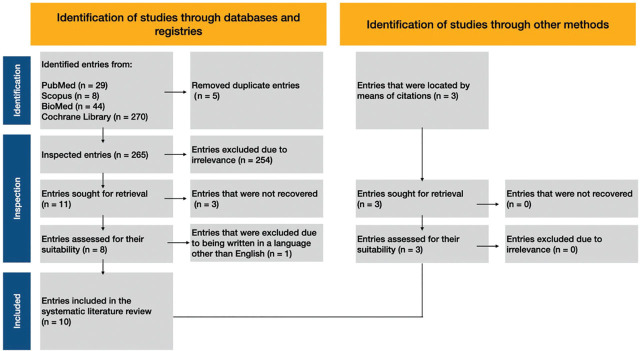
Study selection process.

The PRISMA 2020 flowchart, presented in Fig. 1, illustrates the process of study selection, from initial identification to final inclusion.

### Quality assessment

C.R.A. and D.S independently assessed the quality of the included studies. No discrepancies were found between the evaluators, thus negating the need for a third-party arbitrator. The quality of the selected studies was assessed using the Caldwell framework (9), which is suitable for both quantitative and qualitative research.

### Data extraction

The following data are extracted from the table for this systematic review: (a) First author; (b) Title of each study; (c) Year of publication; (d) Journal: The academic journal in which each study was published; (e) Country: The country where the research was conducted or where the data were gathered; (f) Type of research: The type of research methodology employed, such as quantitative, qualitative, retrospective cohort study, or secondary quantitative analysis; (g) Sample size: The total number of participants in each study; (h) Targeted sample size: The number of women with disabilities or specific subgroups within the total sample; (i) Measurement: The tools and methods used to collect data, such as questionnaires, diagnostic codes, in-depth interviews, and administrative hospital discharge data; (j) Control group: The size and characteristics of the comparison group of women without disabilities; (k) Measured outcome: The main outcomes of interest, such as the assessment of medical outcomes, childbirth experiences, caesarean section rates, postpartum care satisfaction, and hospital readmissions; (l) Key findings; (m) Specific percentages for labour: The breakdown of delivery methods, such as the percentage of caesarean sections or vaginal deliveries; (n) Follow-up; and (o) Limitations of the study.

## Results

This systematic review analysed 10 scientific articles published between January 1, 2010, and April 24, 2024, focussing on the experiences of childbirth and postpartum care for women with disabilities (2, 5, 10,11,12,13,14,15,16,17). Specifically, the review examined three articles that addressed the childbirth experiences of women with sensory impairments (deaf and blind), seven articles focussed exclusively on deaf women, and three articles examined the experiences of blind women. These studies were conducted across a diverse set of countries, including the United Kingdom, Brazil, the United States, England, Ethiopia, and Canada.

Each article was carefully reviewed, and key details were systematically recorded in a Microsoft Excel spreadsheet, adhering to the methodological framework described in the theoretical background. The studies were organised chronologically by publication date, providing a structured approach to analysing the evolution of research on this topic (Table 2).

**Table 2.** Summary of key points from the reviewed studies.

**No.**	**First Author**	**Title**	**Year of publication**	**Country**	**Type of Research**	**Sample Size**	**Targeted Sample Size**	**Measurement**	**Control Group**	**Measured Outcome**	**Key Findings**	**Specific Percentages for Labor (Natural or C-Section)**	**Follow up**	**Limitation of the Study**
**1.**	Redshaw Maggie	Women with disability: the experience of maternity care during pregnancy, labour and birth and the postnatal period (10)	2013	United Kingdom	Secondary quantitative analysis.	A total of 24,155 women participated to the study.	Women with disabilities constituted 6.14% of the sample. Among them, 197 had sensory disabilities.	A questionnaire was distributed, covering various sections related to prenatal care, childbirth, postpartum care, accessibility, information, communication, and birth choices.	The control group consisted of 22,313 women without any disabilities.	The study assessed knowledge, beliefs, and medical outcomes among mothers with and without disabilities, focusing on the availability of services, communication, participation in decision-making, satisfaction, and breastfeeding rates.	In general, women with disabilities had similar access to healthcare during the early stages of their pregnancy compared to those without disabilities. However, they experienced more prenatal check-ups, ultrasounds, cesarean sections, and longer hospital stays, while being less likely to breastfeed. Despite these minor disparities, women with disabilities were often more dissatisfied with the communication and support they received.	Regarding the type of delivery, 24.4% of deaf women underwent a cesarean section. It is noteworthy that women with sensory impairments were equally likely to have a spontaneous vaginal delivery (54.3%) compared to women without disabilities.	Follow-up with the sample 3 months postpartum.	The study’s limitations include its reliance on self-reported data and the fact that no data was collected on socioeconomic status.
**2.**	Werner Heron	Maternal-fetal attachment in blind women using physical model from three-dimensional ultrasound and magnetic resonance scan data: six serious cases (11)	2015	Brazil	Qualitative study.	A total of 6 blind women participated to the study.	A total of 6 blind women participated to the study.	A 3-question questionnaire was distributed to assess the mother-fetus relationship.	No control group.	The connection between the mother and fetus (MFA) was investigated in six blind pregnant women using physical models derived from three-dimensional (3D) ultrasound and magnetic resonance imaging (MRI) data.	The study’s conclusions suggest that cesarean deliveries are predominant, likely due to the structure of private maternity clinics. This implies that the choice of cesarean section is not always driven by medical necessity.	All six women in the sample delivered via cesarean section.	There was no follow-up with the sample.	The limitations of the study include the small sample size and the lack of ability to compare the experiences of the sample.
**3.**	Darney G Blair	Primary cesarean delivery patterns among women with physical, sensory, or intellectual disabilities (5)	2017	United States of America	Quantitative retrospective cohort study of all births in California from 2000 to 2010 using linked birth certificates and hospital discharge data.	The sample included 4,610,955 births. According to the study’s definitions and criteria for the International Classification of Diseases, 9th Revision (ICD-9) codes, 20,894 births (0.45% of the total) were associated with women having a diagnosis code related to potential disability.	The specific sample size included 1,772 deaf women and 1,128 blind women.	Diagnostic codes from the International Classification of Diseases, 9th Revision (ICD-9) were used to identify women with physical, sensory, and intellectual/developmental issues at the time of birth. The diagnoses were derived from hospital discharge data and linked with birth certificate data.	The comparison sample consisted of 4,590,061 births from women without any disabilities.-	The aim of the study was to examine how disabilities affect pregnancy outcomes and to provide information for improving healthcare for women with disabilities during pregnancy and childbirth.	In general, women with disabilities had a primary cesarean section rate of 32.7%, nearly double that of women without disabilities, who had a rate of 16.3%.	Specifically, approximately 22% of the deaf women in the sample of 1,772 underwent cesarean sections. On the other hand, about 43% of the sample of 1,128 women with sensory disabilities had cesarean sections.	The study did not include any follow-up component with the sample.	The limitations of the study include the use of administrative data and, more specifically, the possibility of errors or omissions in the coding of women’s disabilities, as well as the lack of follow-up or re-contact with the sample.
**4.**	Malouf Reem	Access and quality of maternity care for disabled women during pregnancy, birth and the postnatal period in England: data from a national surveυ. (12)	2017	England	This is a secondary analysis and constitutes quantitative research	The sample consisted of 20,094 women who participated in the study. Of these, 1,958 women self-identified as individuals with disabilities, which is approximately 9.5% of the total sample. The remaining 18,136 women did not report having a disability	From the total sample, 174 women had a sensory disability.	The research tool consisted of a structured questionnaire designed to collect national data on postpartum care experiences in England	The control group concerned 18,136 women without disabilities.	The study assessed the childbirth and caregiving experiences of women with disabilities compared to those of women without disabilities in England.	Women with physical disabilities had a lower rate of vaginal deliveries (47.7%) compared to women without disabilities (59.6%). Women with physical disabilities were more likely to undergo a planned cesarean section (18.4%) compared to women without disabilities (11.0%). Women with physical disabilities also had a higher rate of emergency cesarean sections (18.6%) compared to women without disabilities (14.4%).	In general, women with disabilities had a primary cesarean section rate of 32.7%, nearly twice that of women without disabilities, who had a rate of 16.3%.	The study did not include any component of recontacting the sample.	The limitations of the study include reliance on self-reported data, which may affect accuracy due to recall bias.
**5.**	Schiff A. Mellisa	Pregnancy Outcomes Among Deaf Women in Washington State, 1987–2012 (13)	2017	United States of America	It is a retrospective cohort study. It is quantitative research, although it includes qualitative aspects such as comments and analyses.	The study focused on 645 deaf women.	The total sample consisted of 645 deaf women	The data for the study originated from the examination and analysis of official birth and fetal death records in the state of Washington and their subsequent linkage with hospital discharge records. Further analysis was conducted using Poisson regression.	The control group of the study consisted of 6,453 women with no hearing problems. This group was randomly selected to provide a comparison with the 645 deaf women included in the study.	The aim of the study was to evaluate births and overall outcomes for deaf women in the state of Washington.	Deaf women had a slightly increased risk of cesarean section compared to non-deaf women. The relative risk (RR) for cesarean section among deaf women was 1.15. The majority of deaf women (70.1%) had vaginal deliveries, as did non-deaf women (74.4%), but deaf women had longer hospital stays.	Specifically, 29.9% of deaf women delivered via cesarean section, compared to 25.6% of non-deaf women.	The study did not include any component of recontacting the sample	The study’s limitations included potential underestimation and inaccuracies in the diagnosis of deafness, a small sample size, lack of information regarding the severity of hearing loss, unmeasured factors affecting the risk of cesarean section, potential impact of communication difficulties, and the effect of changes over a long data collection period.
**6.**	Mitra Monika	Pregnancy, Birth, and Infant Outcomes Among Women Who Are Deaf or Hard of Hearing (14)	2020	United States of America	It is a retrospective cohort study. It is quantitative research,	The total sample size of the study is 1,188,676 deliveries.	The sample size of deaf or hard-of-hearing women in the study was 1,385.	The data were obtained from the Massachusetts PELL (Pregnancy to Early Life Longitudinal) system. The data were subjected to quantitative analysis. The authors specifically used Chi-square tests and t-tests to compare categorical and continuous variables, respectively, and Poisson and multinomial logistic regressions to compare the risk of adverse pregnancy and birth outcomes between deliveries in deaf and non-deaf women.	The control group consisted of 1,187,291 deliveries from women without any disabilities.	The study compared the outcomes of chronic health conditions, pregnancy-related complications, labor complications, and birth outcomes for deaf or hard-of-hearing women with those of hearing women, while taking into account various demographic and socioeconomic factors.	In general, it was found that deaf or hard-of-hearing women are at increased risk for chronic conditions, pregnancy-related complications, and adverse birth outcomes.	Regarding deliveries, the study found that 32.1% of deaf or hard-of-hearing women had cesarean sections, compared to 28.7% of women without disabilities, while 67.9% of deaf women had vaginal deliveries, compared to 71.3% of women without disabilities. However, the higher likelihood of cesarean section among deaf women was not statistically significant after adjusting for the model.	The study did not include any component of recontacting the sample	The study’s limitations include the lack of clinical and audiometric confirmation of the deaf women’s condition and the absence of data on the communication methods between healthcare providers and deaf women.
**7.**	Mitra Monika	Pregnancy and Neonatal Outcomes Among Deaf and Hard of Hearing Women (15)	2021	United States of America	It is a retrospective cohort study. It is quantitative research,	The total sample size regarded 8,027,938 deliveries	The sample of deaf women consisted of 5,258 individuals.	The study used data from the National Inpatient Sample (NIS) of the Healthcare Cost and Utilization Project (HCUP). It employed weighted Poisson regression models and multivariable regression models within the STATA computing environment.	The control group consisted of 8,022,680 women without disabilities.	The aim of the study was to compare pregnancy complications and neonatal outcomes between deliveries in women with and without deafness based on national hospital discharge data.	Deaf women had a higher likelihood of delivering via cesarean section compared to non-deaf women. Additionally, deaf women exhibited an increased risk of adverse pregnancy outcomes and chronic medical conditions.	It was found that 35.4% of deliveries among deaf or hard-of-hearing women involved cesarean sections, compared to 32.7% of women in the control sample. The proportion of vaginal deliveries for deaf women was 64.6%, while for women without disabilities it was 67.3%.	The study did not include any component of recontacting the sample	The study’s limitations include potential conservative bias due to incomplete identification of deaf and hard-of-hearing women in hospital records, limited accuracy of coded diagnoses and procedures, lack of detailed reproductive health characteristics in the HCUP-NIS data, inability to establish causation due to its cross-sectional nature, and inability to link maternal and infant hospital records.
**8.**	Wudneh Aregahegn	Obstetric violence and disability overlaps: obstetric violence during child birth among womens with disabilities: a qualitative study (16)	2022	Ethiopia	This is a qualitative descriptive phenomenological study.	The total sample size of this study was 22 women with disabilities.	The study included 2 women with disabilities related to deafness. Additionally, it comprised 8 women with sensory disabilities, specifically blindness or partial vision.	The study used in-depth, semi-structured interviews. The interviews were recorded, transcribed verbatim, and analyzed using NVivo software.	The study did not include a control group.	The aim of the study was to document and analyze experiences of obstetric violence among women with disabilities during childbirth. These experiences were categorized into five main themes: physical abuse, verbal abuse, stigma and discrimination, neglect and abandonment, and violations of privacy.	Almost all participants experienced physical abuse, such as slaps and pinches, while a significant number reported verbal abuse, including yelling and insults. Neglect and abandonment were common, with many being left alone during labor. Stigma and discrimination were also widespread, including instances of isolation. Reports also noted extensive breaches of privacy, exposing many participants to onlookers during delivery. Lastly, one woman was forced to undergo sterilization during a cesarean section.	Out of the 22 women in the sample, 16 delivered via cesarean section, which corresponds to 72.72%.	The study did not include any component of recontacting the sample	The study’s limitations include the small sample size and the limited geographic area, as well as the qualitative nature of the data, which restricts the generalizability of the findings.
**9.**	McKee S. Kimberly	Postpartum Hospital Readmissions Among Massachusetts Women Who are Deaf or Hard of Hearing (17)	2023	United States of America	This is a retrospective cohort study. It involves quantitative research	The total sample of the study was 1,385,003 women.	The study included 3,564 deaf women.	The authors analyzed data from the Massachusetts Pregnancy to Early Life Longitudinal Data System from 1998–2017 using Cox proportional hazards models.	The study used a comparison sample of 1,381,439 women without disabilities.	The aim of the study was to compare the risk of postpartum hospitalization and the main indications for postpartum admissions between deaf or hard-of-hearing women and women without disabilities.	Deaf women had a significantly higher risk of hospital admission during all postpartum periods compared to women without disabilities. They also had nearly seven times the risk of repeated hospital admissions within 43–90 days postpartum and nearly four times the risk within 91–365 days postpartum. The most frequent causes of readmission among deaf women were complications of the puerperium/hemorrhage and soft tissue disorders.	Specifically, about 70% of deaf women had a vaginal delivery. This rate is comparable to that of women without disabilities, which is approximately 71%. This means that 30% of women with hearing impairments underwent a cesarean section, a rate that is roughly the same as the 29% of women without disabilities.	There was follow-up with the sample within a year to determine if the women in the sample were admitted to the hospital during the postpartum period.	The study’s limitations related to sample selection. Specifically, these included the focus on broad postpartum care rather than specific visits, the limited inclusion of participants with various disabilities, the under-representation of those requiring extensive accommodations, and a sample that was biased towards white, educated, urban residents.
**10.**	Tarasoff A. Lesley	Unmet needs, limited access: A qualitative study of postpartum health care experiences of people with disabilities (2)	2023	Canada	This is an empirical research study that uses qualitative methods (semi-structured interviews)	The general sample of the study consisted of 31 individuals with disabilities who had recently given birth.	From the sample size, 2 women were blind and 5 were deaf.	The study used semi-structured interviews for data collection. These interviews were guided by a specific set of questions but allowed participants to discuss their experiences in depth. The interviews covered the chronological sequence of the participants’ most recent experiences with perinatal care, from pregnancy to the postpartum period, focusing on the types of services they accessed, whether the services met their needs, and recommendations for improving care. The data were analyzed using thematic analysis.	The study did not include a control group.	The study focused on the experiences and perceptions of women with disabilities regarding the care they received postpartum. The components of the study included (i) the adequacy of postpartum care, (ii) the accommodations provided for the women’s disabilities, (iii) whether they experienced fear of being judged and discrimination, and (iv) the accessibility and overall quality of formal services.	The key findings of the study are that the majority of participants reported inadequate physical recovery care postpartum and encountered significant gaps in postpartum care, accommodations for people with disabilities, and mental health support.	48.38% of the participants had delivered by cesarean section.	The study did not include any component of recontacting the sample	The study’s limitations include the absence of a control group, reliance on self-reported data, limited diversity within the sample, underrepresentation of certain disabilities, focus solely on formal services, lack of long-term follow-up, broad focus of the interview guide, and limited ability to generalize regarding intersections with other forms of discrimination and the impact of poverty.

### Caesarean section rates

Across all studies, women with disabilities demonstrated an increased likelihood of caesarean section deliveries compared to women without disabilities. This trend was consistent irrespective of the nature of the disability, whether physical, sensory, or intellectual. For instance, in the study by Darney et al. (5), women with disabilities had a caesarean section rate of 32.7%, nearly double that of women without disabilities (16.3%). In a study conducted by Redshaw et al. (10), 24.4% of women with sensory disabilities underwent caesarean sections, while Malouf et al. (12) reported that women with physical disabilities had a higher incidence of both planned (18.4%) and emergency (18.6%) caesarean sections compared to their non-disabled counterparts (11.0% and 14.4%, respectively).

This pattern persisted even in smaller-scale studies, such as Werner et al.’s (11) study of blind women in Brazil, where all six participants delivered via caesarean section. Similarly, in the qualitative study by Wudneh et al. (16) focussing on obstetric violence in Ethiopia, 72.72% of women with disabilities delivered via caesarean section. Studies focussing on deaf women, such as those by Schiff et al. (13) and Mitra et al. (15), also reported increased caesarean rates (29.9% and 35.4%, respectively), though the increase was not statistically significant after adjustment in some instances (14).

### Vaginal deliveries

Despite the higher rates of caesarean sections, vaginal deliveries remained common among women with disabilities in several studies. Redshaw et al. (10) found that women with sensory disabilities had a spontaneous vaginal delivery rate of 54.3%, comparable to that of women without disabilities. Mitra et al. (14) reported that 67.9% of deaf women delivered vaginally, which was not significantly different from the 71.3% rate observed in women without disabilities.

### Maternal–fetal attachment and postpartum experiences

The qualitative study by Werner et al. (11) provided insight into maternal–fetal attachment among blind women using three-dimensional ultrasound and magnetic resonance imaging (MRI) data. This study highlighted that the connection between the mother and fetus was influenced using physical models, and the findings suggested that caesarean deliveries were often chosen due to the structure of private maternity clinics rather than medical necessity. Similarly, Tarasoff et al. (2) highlighted significant gaps in postpartum care for women with disabilities, with many participants reporting inadequate physical recovery care and limited accommodations for their disabilities.

### Healthcare access and satisfaction

Several studies noted disparities in healthcare access and satisfaction among women with disabilities. Redshaw et al. (10) found that while women with disabilities had similar access to healthcare during the early stages of pregnancy, they experienced more prenatal checkups, ultrasounds, and longer hospital stays. However, they were less likely to breastfeed and expressed greater dissatisfaction with communication and support during childbirth and postpartum care. Similar concerns were echoed by Wudneh et al. (16), who reported widespread experiences of obstetric violence among disabled women, including physical and verbal abuse, neglect, and breaches of privacy.

### Postpartum hospital readmissions

McKee et al. (17) explored postpartum hospital readmissions among deaf women in Massachusetts. The study found that deaf women had a significantly higher risk of hospital readmissions during all postpartum periods than women without disabilities. Notably, deaf women had nearly seven times the risk of repeated hospital admissions within 43–90 days postpartum and nearly four times the risk within 91–365 days postpartum.

### Limitations of the studies

The studies reviewed presented various limitations, including reliance on self-reported data, potential recall bias, small sample sizes, and the absence of long-term follow-up. Several studies, such as those by Redshaw et al. (10) and Malouf et al. (12), relied on self-reported data, which may have affected the accuracy of findings due to recall bias. The small sample sizes in studies like Werner et al. (11) and Wudneh et al. (16) limited the generalisability of their findings, as did the qualitative nature of some studies. Additionally, many studies did not re-contact participants for follow-up, limiting the ability to assess long-term outcomes for women with disabilities after childbirth.

### Obstetric violence and discrimination

Wudneh et al. (16) provided important qualitative data on the experiences of obstetric violence among women with disabilities in Ethiopia. The study highlighted that most participants experienced physical and verbal abuse during childbirth, neglect, and discrimination. Similarly, Tarasoff et al. (2) noted that many participants felt fear of being judged and faced discrimination during their postpartum care, underscoring the need for more inclusive and supportive maternity care for women with disabilities.

## Discussion

Women with disabilities, across various studies, were more likely to undergo caesarean sections, experience longer hospital stays, and report dissatisfaction with communication and support during childbirth. Additionally, some studies identified specific risks for deaf or hard-of-hearing women, including higher rates of postpartum hospital readmissions and adverse birth outcomes. Despite these disparities, many women with disabilities were able to deliver vaginally, indicating that caesarean sections were not universally necessary for this population. However, the findings across studies consistently highlight the need for better communication, improved support, and tailored accommodations to enhance the childbirth and postpartum experiences of women with disabilities.

Another literature review examined the childbirth experiences of women with physical disabilities, focussing on barriers such as healthcare professionals’ lack of knowledge, negative attitudes, and inaccessible facilities (18). The review highlighted the challenges in delivery methods, pain management, and communication, emphasising the need for improved clinician training, better collaboration, and more inclusive care environments to ensure positive outcomes. Compared to our review on women with sensory disabilities, both studies highlight similar healthcare system deficiencies, but our review found higher caesarean rates, possibly due to unique communication barriers.

Another review focussed on the perinatal care experiences of women with vision disorders, highlighting the lack of healthcare staff training and inadequate facilities tailored to these women’s needs (19). The review identified barriers such as dissatisfaction with the quality of care, unsuitable antenatal classes, and the stigmatisation of motherhood among women with visual impairments. The authors emphasised the need for specialised training for healthcare providers to better accommodate these women’s functional needs and improve their maternity care experience (19). In comparison, our review on women with sensory disabilities (deaf or blind) also found higher rates of caesarean sections, but these were often attributed to communication gaps and healthcare providers’ assumptions about the women’s ability to give birth vaginally. While both reviews emphasise inadequate provider training and systemic healthcare barriers, our review highlights a broader issue of communication challenges, particularly for deaf women, which often led to misunderstandings about care options and outcomes. Additionally, our review suggests a more pronounced lack of informed decision-making due to these communication difficulties, a factor less emphasised in the vision disorder review. Both reviews stress the importance of tailored, inclusive care, but the underlying causes of inadequate care differ slightly based on the type of sensory impairment.

Discrimination against these women is a pervasive issue, with alarming instances of human rights violations, such as cases where deaf women were coerced into sterilisation under the pretext of undergoing a caesarean section (16). Such actions are not only reprehensible but also violate bodily autonomy, eroding trust between patients and healthcare providers. Ensuring that healthcare is delivered in a manner that respects patient autonomy and informed consent is critical to maintaining ethical standards and safeguarding the dignity of all individuals (16).

Communication barriers significantly exacerbate the difficulties faced by deaf and blind women during their interactions with the healthcare system. These barriers often hinder their ability to access adequate prenatal and postnatal care, and they may struggle to communicate critical concerns regarding their children’s development (2,13). The inability to communicate effectively with healthcare providers limits their participation in decision-making processes, compromising the quality of care they receive. To address these issues, healthcare systems must prioritise inclusivity and ensure that the unique needs of women with sensory disabilities are met.

In line with the Sustainable Development Goals (SDGs) aimed at promoting inclusion and reducing inequalities, it is imperative to develop healthcare systems that accommodate the diverse needs of patients with disabilities. This can be achieved through several strategic actions. One key solution is investing in assistive communication technologies and appointing specialised sign language interpreters with knowledge of medical terminology to bridge the communication gap. Additionally, healthcare professionals should receive specialised training in effective communication methods tailored to individuals with hearing and vision impairments (20). Awareness and understanding of disability among healthcare professionals are crucial for improving care delivery. Disabilities are often misunderstood, and individuals with disabilities may experience their condition differently from how those without disabilities perceive it (21). Certified training programs and awareness seminars can help medical staff better understand the specific needs of women with disabilities, fostering a more inclusive and compassionate healthcare environment. Furthermore, the creation of support networks dedicated to mothers with disabilities during pregnancy, childbirth, and postpartum care is essential to ensuring comprehensive care (22).

Personalising the childbirth experience is another promising approach. For example, creating delivery rooms designed with accessibility in mind—such as installing support rails and guiding paths for blind women—can greatly improve the safety and comfort of the birthing environment. Technological innovations that facilitate communication and information sharing can also enhance the overall care experience for women with sensory disabilities (23).

Ethically, healthcare providers are obligated to respect patients’ preferences and decisions. Incorporating practices that align with women’s choices is fundamental to ensuring their autonomy and fostering a positive childbirth experience (22, 24). This principle is embedded in the broader concept of informed decision-making and respect for individual autonomy. Specialised medical staff, particularly midwives trained to provide tailored care for women with disabilities, can play a vital role in creating a safe and supportive environment for both mothers and their families (24).

This systematic review has several limitations that affect the breadth and depth of its findings. First, many of the included studies had small sample sizes, particularly those focussing on women with sensory disabilities, which limits the generalisability of the results. These smaller studies may not accurately reflect the experiences of the broader population of women with sensory impairments. Additionally, most of the studies were observational in nature, and many relied on self-reported data, introducing the possibility of recall bias. The lack of randomised controlled trials or more robust longitudinal studies further weakens the ability to establish causal relationships between sensory disabilities and specific childbirth outcomes.

Another limitation is the geographical and cultural variation in the studies. The research was conducted across different countries, each with distinct healthcare systems, policies, and cultural attitudes toward women with disabilities, making it challenging to generalise the findings universally. Furthermore, long-term follow-up data were lacking in most studies, limiting the understanding of postnatal care and long-term maternal and child health outcomes. Issues such as obstetric violence and discrimination, although mentioned in some studies, were often underreported or not fully explored, suggesting a possible underestimation of their prevalence. Finally, the exclusion of non-English language studies may have limited the scope of the review by omitting potentially relevant findings from other regions. These limitations highlight the need for more comprehensive research with larger, more diverse populations, improved study designs, and a greater focus on postnatal care and long-term outcomes.

## Conclusions

In conclusion, this systematic review revealed several critical insights into the childbirth experiences of women with sensory disabilities, particularly deaf and blind women. The analysis found a higher prevalence of caesarean deliveries among this demographic, influenced by medical necessity, biases from healthcare professionals, or personal preferences. Despite this, many women with disabilities are fully capable of having successful vaginal deliveries, underscoring the importance of promoting information about the benefits of natural childbirth. Additionally, the review highlighted significant deficiencies within the healthcare system, including inadequate communication, lack of appropriate infrastructure, and insufficient information tailored to the needs of women with sensory disabilities.

To address these issues, healthcare systems should adopt a framework that fosters inclusion, equity, and respect for patient autonomy. Bridging the communication gap between patients and healthcare providers, as well as providing proper support and resources, will enhance the quality of care and improve trust in the patient–provider relationship. Ultimately, by ensuring that women with sensory disabilities receive dignified and equitable care, we can reduce healthcare disparities and promote better outcomes for both mothers and their newborns. Further research and the development of inclusive policies are essential steps toward achieving these goals.

## Abbreviations

MRI: magnetic resonance imaging
PICOST: Population, Intervention, Comparison, Outcome, Study design, Timing
PRISMA: Preferred Reporting Items for Systematic Reviews and Meta-Analyses
SDGs: Sustainable Development Goals
WHO: World Health Organization.
